# Effects of Sericea Lespedeza Supplementation on Steers Grazing Wild-Type Endophyte-Infected Tall Fescue

**DOI:** 10.3390/ani15030373

**Published:** 2025-01-28

**Authors:** Sanjok Poudel, Gabriel J. Pent, John H. Fike, Wayne E. Zeller, Brittany E. Davis

**Affiliations:** 1 Cooperative Extension, College of Agriculture and Environmental Sciences, North Carolina A&T State University, Greensboro, NC 27411, USA; 2 Shenandoah Valley Agriculture Research and Extension Center, Virginia Tech, Raphine, VA 24472, USA; gpent@vt.edu; 3 School of Plant and Environmental Sciences, Virginia Tech, Blacksburg, VA 24060, USA; jfike@vt.edu; 4 US Dairy Forage Research Center, USDA-ARS, Madison, WI 53706, USA; wayne.zeller@usda.gov; 5 Forage Animal Production Research Unit, Agricultural Research Service, United States Department of Agriculture, Lexington, KY 40546, USA; brittany.davis@usda.gov

**Keywords:** tall fescue, alkaloids, endophyte, condensed tannins, lespedeza

## Abstract

Condensed tannins (CTs) in certain leguminous forages can mitigate toxic alkaloid absorption linked to fescue toxicosis due to their high affinity towards various steroidal and protein-like alkaloids. However, their use as feed supplements remains underexplored. This study evaluated the impact of CT-rich sericea lespedeza (*Lespedeza cuneata*) pellets on the post-ingestive effects of fescue toxicosis. Twelve steers on wild-type endophyte-infected tall fescue pastures received either sericea lespedeza pellets (LES) or LES with polyethylene glycol (LPEG; negative control) for 12 weeks over three consecutive summers. Body weight, hair coat scores, temperatures (rectal and extremity), cortisol levels, and caudal artery lumen area were measured every four weeks. Steers fed LES showed trends toward higher ADG (*p* = 0.0999) and reduced hair retention (*p* = 0.0547) compared to those fed LPEG. Steers on LES also showed hotter tail skin temperatures (*p* = 0.0053) and cooler rectal temperatures (*p* < 0.0001) compared to those fed LPEG. LES-fed steers had a 21% larger caudal artery lumen area (*p* < 0.01), suggesting reduced vasoconstriction. Additionally, LES-fed steers tended to have lower hair cortisol (*p* = 0.0746), indicating reduced chronic stress. These results suggest that supplementation with CTs may alleviate the post-ingestive effects of fescue toxicosis, potentially by improving blood flow and reducing stress. However, further research is needed to determine whether CTs directly reduce alkaloid absorption, as well as to validate the long-term efficacy of CT supplementation.

## Simple Summary

Fescue toxicosis caused by wild-type endophyte-infected (WE) tall fescue (TF) is a major issue among livestock producers in the southeastern US. This study explores the use of condensed tannin-based diet supplements in reducing the post-ingestive effects of fescue toxicosis. Twelve steers on WE pasture were supplemented with either sericea lespedeza pellets (LES; a source of condensed tannins) or LES mixed with polyethylene glycol (LPEG) as a control treatment for 12 weeks over three consecutive summers. Steers on LES tended to have higher average daily gain and slicker hair coats compared to steers on control treatment. Steers fed LES had hotter tail skin temperatures and cooler rectal temperatures than those fed the control diet. These steers also exhibited lower stress levels and a 21% larger caudal artery lumen, thus indicating improved blood circulation. These findings suggest that the condensed tannin-based diet supplement may be a valuable tool for managing fescue toxicosis in cattle. However, more research is needed to determine whether the supplement can reduce the absorption of toxic alkaloids in tall fescue, as well as to understand the long-term efficacy of supplementing a condensed tannin-based diet to cattle.

## 1. Introduction

Fescue toxicosis, resulting from toxic alkaloids in wild-type endophyte-infected (WE; *Epichloë coenophiala*) tall fescue (*Schedonorus arundinaceus*), poses significant challenges and leads to nearly USD 2 billion in annual economic losses for the US beef industry [1]. This is probably an underestimate given that this assessment was conducted about a decade ago. As WE tall fescue is the predominant cool-season pasture forage in the southeast and the north–south transition zone, livestock producers in these regions of the US would gain significant benefits by determining the most effective mitigation strategy to deal with fescue toxicosis.

Various strategies have been explored to reduce the harmful effects of livestock consuming WE tall fescue, including supplementation with vitamin E, thiamine, or protein [2,3,4] and the ammoniation of feedstuffs [5]. However, these strategies require additional handling and expenses. The use of vaccines [6] or dopamine antagonists [7] for fescue toxicosis mitigation has not been practically feasible due to the cost and stress associated with multiple injections of these pharmacological substances. Changing WE tall fescue pastures to novel endophyte-infected (NE) tall fescue is often the best option to deal with fescue toxicosis. These improved cultivars have nutritive values comparable to or greater than those of ‘Kentucky 31’, the predominant cultivar of WE tall fescue, but the associated endophytes have no deleterious impacts on livestock. Past studies have reported animals grazing NE tall fescue to have greater ADG and serum prolactin levels, along with lower core body temperatures, compared to animals on WE tall fescue [8,9]. Renovating pastures with non-toxic tall fescue or alternative grass species is typically costly, with expenses of around USD 600 per hectare in 2015 [1]. Additionally, this conversion may not be practical in cases of uncertain land leases or highly erodible land, and many farmers face difficulties due to the loss of production during the transition. Additionally, the economic costs of pasture renovation may take up to three years post-renovation to recoup [10]. Thus, there remains a need to identify economically viable and practical solutions for dealing with fescue toxicosis in livestock.

One potential approach to address fescue toxicosis is the inclusion of feed supplements that can hinder the absorption of toxic alkaloids within the digestive system, thereby counteracting its effects. Tannins represent a diverse group of polyphenolic polymers synthesized by plants in response to various physiological demands and stressors [11]. These compounds can be categorized as condensed tannins (CTs) and hydrolyzable tannins (HT) [12]. Certain forages, trees, and shrubs produce CTs as a defense against herbivores [13]. Integrating CT-rich forages into the diets of ruminants can yield both advantageous and disadvantageous outcomes [14]. Including CT-containing forages in the diet can improve animal performance directly, by controlling anthelmintic-resistant gastrointestinal parasites [15], and indirectly, by enhancing escape protein supply [15,16] along with improving nitrogen-use efficiency [17] and decreasing methane production [18]. CTs’ strong protein-binding affinity shields proteins from degradation by rumen microbes, enhancing protein availability [19]. However, excessive CT intake can yield adverse effects, such as reductions in palatability, dry matter intake, and the digestibility of essential nutrients like protein, carbohydrates, and fats, thereby compromising feed efficiency and animal productivity [20,21].

Along with protein, CTs can also bind with various steroidal and protein-like alkaloids [22]. The inclusion of CT-producing forage species in pasture may reduce the symptoms of fescue toxicosis by binding to these toxic alkaloids. The CTs may help reduce the absorption of alkaloids through the gastrointestinal epithelia, thus mitigating their toxic effects [23]. Various studies have reported increased intake of and preference for WE tall fescue along with changes in various physiological responses of animals when supplemented with legumes rich in CTs [24,25,26,27]. This could be attributed to the inactivation of alkaloids through binding with CTs in the gastrointestinal tract, mitigating negative effects on intake and digestibility. The formation of stable tannin–alkaloid complexes results in their excretion in the feces [28].

Sericea lespedeza [{*Lespedeza cuneate* (Dum. Cours.) G. Don}; SL], a warm-season perennial legume well suited to the southern US climate [29], boasts a high CT concentration that, in some cases, may have negative effects on grazing livestock. However, cultivars like ‘AU Grazer’ with lower CT levels have been developed, which do not adversely affect livestock. This cultivar of SL is commonly used as an alternative to anthelmintic drugs for controlling gastrointestinal parasites in small ruminants [30,31,32,33,34]. Given the likely interaction of CTs with ergot alkaloids, the ingestion of SL may result in a binding of the toxic alkaloids present in tall fescue. Pelletizing SL for feed supplementation could further enhance the utility of this forage by improving convenience in handling, storing, and feeding. However, the effectiveness of pelleted SL as a feed supplement in reducing the impact of fescue toxicosis within a grazing system remains to be established.

The objective of this study was to assess the potential benefits of incorporating feed supplements rich in CTs, such as SL pellets, to counteract the effects of fescue toxicosis in steers grazing WE tall fescue. Our preliminary laboratory analysis showed that when an aqueous solution of purified CTs from sericea lespedeza pellets at 4 mg/mL (400 μL) was added to 400 μL of a saturated aqueous solution of ergotamine tartrate, the CTs interacted with ergot alkaloids, precipitating them from the solution. Based on these findings, we hypothesized that steers grazing WE tall fescue and supplemented with CTs (via SL pellets) would exhibit reduced symptoms of fescue toxicosis, including increased hair shedding, greater average daily gain, reduced vasoconstriction, and lower stress levels, compared with steers receiving similar supplementation but without CT.

## 2. Materials and Methods

### 2.1. Experimental Site

This 3-year summer grazing trial was conducted from late June to mid-September, 2021 to 2023, at Virginia Tech’s Shenandoah Valley Agricultural Research and Extension Center (SVAREC) in Raphine, VA (37°56′ N, 79°13′ W). The primary soil type at the site is Frederick silt loam, mixed mesic, and Typic Paleudult.

### 2.2. Weather Data

Daily ambient temperature (AT) and relative humidity (RH) data for the research site were obtained from the Virginia Tech WeatherSTEM Data Mining Tool (https://vt-arec.weatherstem.com/, accessed on 12 December 2023) for the entire study period across all years. The temperature–humidity index (THI) was calculated daily using the AT and RH data according to the equation from Mader et al. [35]:

THI = [(0.8 × AT) + (RH/100) × (AT − 14.4)] + 46.4(1)

### 2.3. Pasture and Forage Analysis

In 2021, endophyte levels were determined by testing 50 randomly selected tillers using the immunoblot test [36]. For alkaloid concentrations, forage samples were collected at 4-week intervals (days 0, 28, 56, and 84), freeze-dried, and ground to pass through a 1 mm screen. These samples were analyzed for total ergot alkaloid concentration using an ELISA test kit (Agrinostics Ltd., Watkinsville, GA, USA). In 2023, vegetation composition was assessed twice by recording plant cover percentages at 30 random points using 0.25 m^2^ quadrats. Vegetation composition data were not recorded for the years 2021 and 2022. Available forage biomass was estimated by harvesting six 0.25 m^2^ quadrats every 4 weeks, drying the samples at 60 °C for 72 h, and calculating the dry weight. Dried samples were ground for nutritive analysis, first through a 2 mm screen and then through a 1 mm screen, and scanned with a NIRS^TM^ DS2500 F (FOSS, Hillerod, Denmark) for estimating crude protein, acid detergent fiber, and neutral detergent fiber concentrations using the mixed grass hay equation [37].

### 2.4. Animal Use and Feed Supplement Treatment

Each summer trial used twelve 7-to-8-month-old, fall-born Angus cross steers from the SVAREC farm herd in Raphine, VA. The initial body weights were 260 ± 6.4 kg (2021), 273 ± 4.1 kg (2022), and 237 ± 3.2 kg (2023). All steers were weaned and raised on pastures primarily consisting of ‘Kentucky-31’ tall fescue. The steers were stratified by body weight and assigned to one of two diet supplements: SL pellets (LES) or SL pellets mixed with polyethylene glycol (LPEG; 50 g/kg DM) to negate the condensed tannins (CTs). Preliminary tests confirmed that the CTs were inactivated in the LPEG treatment, allowing for the separation of tannins and the nutritional effects of lespedeza. The SL pellets (85% SL leaf meal, 15% molasses/lignin binder) were sourced from Sims Brothers, Inc., and a single composite sample from each year was analyzed for chemical composition by Cumberland Valley Analytical Services, Inc. (Waynesboro, PA, USA) by wet chemistry. Separate sets of composite pellet samples were collected across two years: one sample from a single batch in 2021, and three samples from different batches in 2022. These samples were used for the isolation and purification of CTs present in these pellets [38,39]. In 2023, no new samples were collected or analyzed, as the study continued using the same pellets from 2022. These purified CTs were used in their compositional and structural determination, employing two-dimensional nuclear magnetic resonance spectroscopy [39,40]. These highly pure CTs also served as a reference standard in the determination of CT concentrations in these pellet samples using the HCl–butanol–acetone–iron (HBAI) assay, as outlined in [41]. Specific details of the CT purification, structure determination, and CT content analyses are provided in the accompanying Supplementary Materials.

The steers were continuously stocked on a 2 ha pasture, and supplements were provided individually via feed bunks with Calan gates based on body weight (0.5% BW/day). The steers had free access to trace minerals and clean water.

### 2.5. Animal Body Weight and Hair Coat Score

The unshrunk body weight (BW) of the steers was recorded on two consecutive days (days −1 and 0, and days 83 and 84) between 0700 and 0800 h, using a Tru-Test XR5000 scale (Datamars Limited, Auckland, New Zealand), with averages used to determine the initial and final BW. The average daily gain (ADG) was calculated by dividing the total BW gain by the number of days on treatment. Hair coat scores were assessed every 4 weeks using a 5-point scale (1 = complete shedding, 5 = no shedding), as described by Gray et al. [42]. Multiple evaluators scored the hair coat, and the average score per collection date was used throughout the study.

### 2.6. Extremity and Rectal Temperatures

Thermal images of body extremities (ear, front left foot, and tail) were captured every 4 weeks using a FLIR T630SC thermal camera (Teledyne FLIR LLC, Santa Barbara, CA, USA) between 0700 and 0800 h. The animals were held in a shaded area, and images were taken from approximately one meter away. FLIR Research IR Max software (Version 4.40.9.30) was used to calculate the average temperatures of specific areas, including foot regions like the pastern, coronary band, and toes. Rectal temperatures were recorded every 4 weeks using a veterinary thermometer. A similar procedure was used by Poudel et al. [43].

### 2.7. Hair Sample Collection, Cortisol Extraction, and Analysis

Hair samples were collected from steers on day 0 by clipping a 15 cm × 15 cm area on the left rump using an electric clipper (Premier 1 Supplies, Washington, IA, USA). The same site was re-shaved every 4 weeks (days 28, 56, and 84). Samples were wrapped in aluminum foil and stored at room temperature until analysis. Hair cortisol extraction followed the method of Poudel et al. [44], using HPLC-grade methanol. Cortisol concentrations were measured using a commercial ELISA kit (Cayman Chemical, Ann Arbor, MI, USA), with inter-assay and intra-assay CVs of 14.9% and 8.6%, respectively.

### 2.8. Doppler Ultrasonography

The luminal area of the caudal artery was measured using Doppler ultrasound, as described by Aiken et al. [45]. A Classic Medical TeraVet 3000 Ultrasound Unit (Classic Universal Ultrasound, Tequesta, FL, USA) with a 12 MHz linear transducer (12L5-VET) set to a 4 cm depth was used to scan the caudal artery at the 4th coccygeal (Cd4) vertebra. Scans were conducted every 4 weeks during the summer of 2022.

### 2.9. Statistical Analysis

A mixed-effects ANOVA was performed using PROC MIXED in SAS Studio (v. 3.5), with steers as the experimental units. Repeated-measures analysis by period, with a standard variance–covariance structure, was applied to ADG, HRS, hair cortisol, arterial luminal area, and temperature data. Year-by-treatment interaction effects were included in the model for variables where data were collected across multiple years to evaluate potential variability attributable to the interaction of treatment and year. Statistical significance was set at *p* < 0.05, and trends were noted for 0.05 < *p* < 0.10.

## 3. Results

### 3.1. Weather Data

For the study period in 2021, the mean daily air temperature was 22.1 °C, with a corresponding relative humidity (RH) of 76.4%. These values were 27.7 °C and 70.7% RH in 2022, and 22.0 °C with 76.3% RH in 2023. For the three grazing seasons, the total precipitation was 81.3 mm, 76.2 mm, and 98.1 mm, respectively. The daily mean temperature–humidity index (THI) was higher for 2022 (77.9) compared to 2021 and 2023, with a mean THI of 70.0.

**Figure 1:**
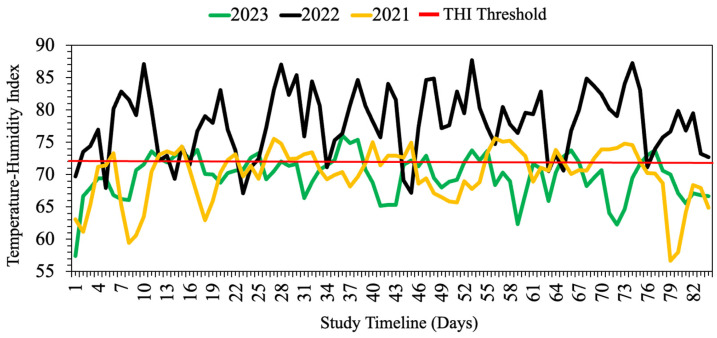
Daily average temperature–humidity index (THI) throughout the study period at the Shenandoah Valley Agriculture Research and Extension Center, Raphine, VA, across three summers.

In 2021, the maximum THI was 75.6, with the average daily THI exceeding the thermoneutral zone (THI = 72) for 34 days (Figure 1). In 2022, the maximum THI reached 87.2, surpassing the thermoneutral zone for 72 days. In 2023, the maximum THI was 76.0, with 22 days above the thermoneutral zone. These results highlight prolonged thermal stress for livestock during all three summers due to frequent THI values above the thermoneutral range.

### 3.2. Forage and Supplement Measures

**Figure 2:**
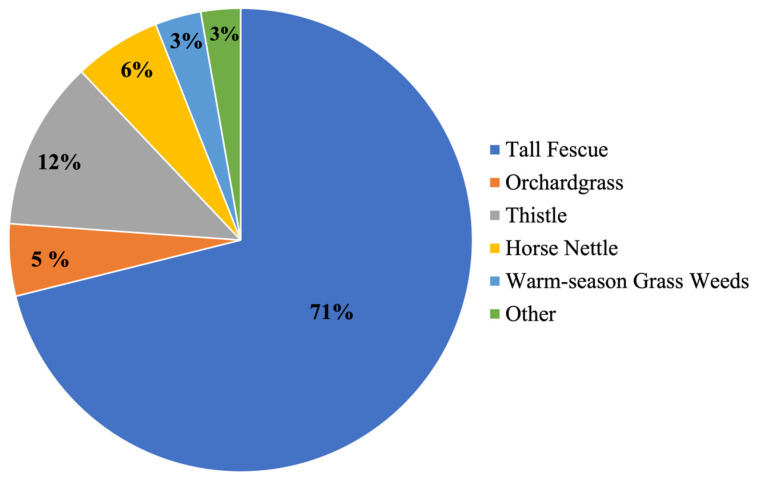
Vegetation composition of the experimental pasture during the summer of 2023 at the Shenandoah Valley Agricultural Research and Extension Center, Raphine, VA, USA.

In 2021, an immunoblot test revealed that the experimental pasture was 100% endophyte-infected. That same year, the average TEA concentration of the experimental pasture was 1650 ppb, while in 2022 and 2023 it was 2330 ppb and 2460 ppb, respectively. Tall fescue (71%) was the predominant forage in the experimental pasture, followed by orchardgrass (*Dactylus glomerata*, 5%), while there was a significant prevalence of broadleaf weeds such as horse nettle (*Solanum carolinense*; 6%) and *Cirsium* spp. (12%). Other vegetation species include red clover (*Trifolium pretense*), white clover (*Trifolium repens*), quack grass (*Elymus repens*), and Kentucky bluegrass (*Poa pratensis*; Figure 2).

The experimental pasture showed consistent levels of available forage biomass and nutritional composition across the three study years (Table 1). Similarly, the lespedeza (LES) diet supplement maintained relatively stable nutritional content over the study period (Table 2). In 2021, the CT concentration averaged 189 g kg^−2^ DM from a single composite sample. In 2022, three separate samples, representing different batches, were analyzed, with CT concentrations of 204, 198, and 212 g kg^−2^ DM, respectively.

### 3.3. Average Daily Gain (ADG) and Hair Coat Score

There was no year × treatment interaction observed in these studies for ADG (*p* = 0.1648) and hair coat score (*p* = 0.0876). Steers in the LES diet supplement treatment tended (*p* = 0.0999; Table 3) to have greater ADG and lower (*p* = 0.0547) hair coat scores compared to steers in the LPEG diet supplement treatment (Table 3).

### 3.4. Extremity and Rectal Temperature

There was no year × treatment interaction observed for ear, hoof, and tail surface temperatures or rectal temperature data (*p* > 0.1230) in these studies. Ear skin and hoof temperatures did not differ (*p* ≥ 0.1826) between the LES and LPEG diet supplement treatments (Table 4). However, the tail skin temperature was hotter (*p* = 0.0053) in LES-fed steers compared to those receiving LPEG, and the rectal temperature of steers supplemented with LES was significantly cooler (*p* < 0.0001) compared to those receiving LPEG diet supplement treatment.

### 3.5. Hair Cortisol Levels

**Figure 3:**
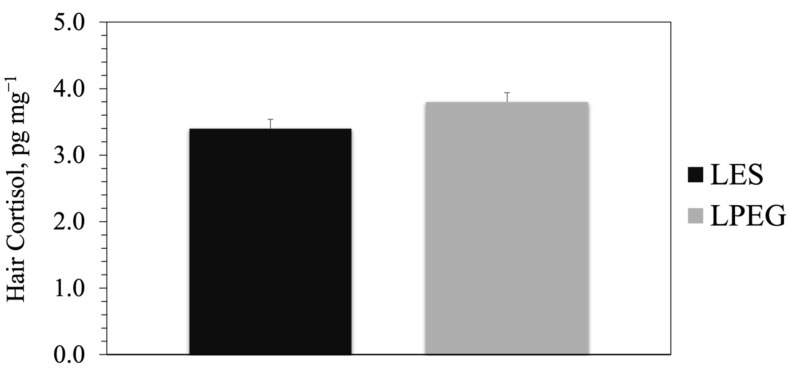
Hair cortisol (pg mg^−1^) concentration of steers on toxic endophyte-infected tall fescue supplemented with either sericea lespedeza pellets (LES) or sericea lespedeza pellets mixed with polyethylene glycol (LPEG) across three summers. No significant year × treatment interaction (*p* = 0.6717). Level of significance: *p* = 0.0746.

No significant year × treatment (*p* = 0.6717) interactions were observed in the hair cortisol data. The hair cortisol level of steers on LPEG diet supplement treatment tended (*p* = 0.0746) to be greater than that of steers on the LES diet supplement (Figure 3).

### 3.6. Doppler Ultrasonography

**Figure 4:**
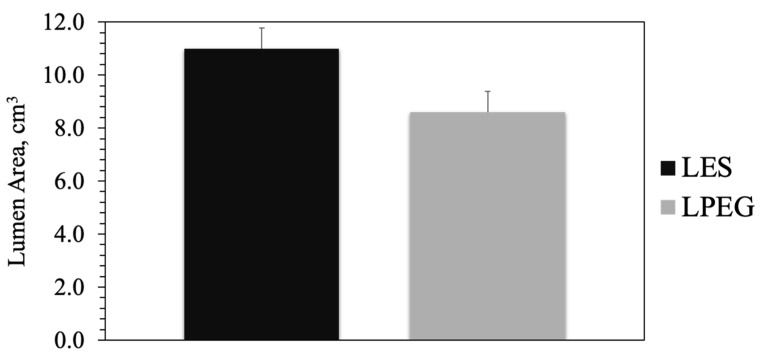
Ultrasonic measures of the lumen area of the caudal arteries (cm^3^) of steers on toxic endophyte-infected tall fescue supplemented with either sericea lespedeza pellets (LES) or sericea lespedeza pellets mixed with polyethylene glycol (LPEG). No significant period × treatment interaction (*p* = 0.2854). Level of significance: *p* < 0.01.

No significant period × treatment interaction (*p* = 0.2854) was detected in the proportionate differences in caudal artery luminal areas. However, steers supplemented with the LPEG diet supplement treatment had smaller (*p* = 0.0063) mean caudal artery luminal areas, which were decreased by an average of 21.2% relative to the LES diet supplement treatment (Figure 4).

## 4. Discussion

The purpose of this study was to investigate the potential of CT-containing feed supplements, such as SL pellets, to reduce the symptoms of fescue toxicosis in steers grazing WE tall fescue, along with their numerous other nutritional benefits. Due to the affinity of CTs for various nitrogenous compounds, diets rich in CTs can increase the supply of rumen-undegradable protein [15,16] and decrease gastrointestinal parasite infestations [46,47], ultimately enhancing overall ruminant productivity. Diets rich in CTs can also enhance nitrogen-use efficiency [17] and reduce methane production in the rumen [18], thereby mitigating the environmental consequences of livestock farming. Including forages that contain CTs in animal diets may help mitigate the post-ingestive effects of fescue toxicosis, as these compounds have an affinity for the steroidal and protein-like alkaloids produced by the endophytic fungus in tall fescue [22,28].

The tall fescue present in the experimental pasture was 100% endophyte-infected, and the total concentration of ergot alkaloids exceeded 1500 ppb in all three summers. These pastures were considered sufficiently toxic to induce symptoms of fescue toxicosis [48]. Animals grazing on endophyte-infected tall fescue with an endophyte infection level exceeding 22% [49] and total ergot alkaloid concentrations greater than 400 ppb [50,51] may display visible signs of fescue toxicosis. In the current study, the total CT concentration in the LES diet supplement was in the range of 189–212 g kg^−1^ DM. The CTs in LES may bind with toxic alkaloids, reducing their absorption through the gastrointestinal epithelia and, thus, minimizing their toxic effects [23]. In this study, LES was mixed with polyethylene glycol at a rate of 50 gm kg^−1^ DM to provide the LPEG supplement. The addition of polyethylene glycol forms complexes with CTs, effectively deactivating them [52,53,54]. This provides an opportunity to assess whether the effects attributed to feeding LES are due to the presence of CTs or the nutritional effects of the supplement. It is also important to note that excessive dietary CT intake could lead to negative effects on livestock [21]. This introduces uncertainties regarding how to harness the positive benefits of CTs while avoiding potential negative effects associated with excessive concentrations.

Steers on the LES supplement treatment tended to have greater ADG compared to steers on LPEG. Several studies have reported improved intake of and preference for WE tall fescue in animals supplemented with forage legumes rich in CTs, such as birdsfoot trefoil (*Lotus corniculatus*) and sainfoin (*Onobrychis viciifolia*) [24,25,26,27]. The increased gain observed in animals on LES might also be attributed to a greater supply of rumen-undegraded protein, as CTs possess a strong binding affinity for proteins, safeguarding them from rumen microbial degradation [19]. The steers fed the CT action-negating, polyethylene glycol-containing LPEG supplement experienced the post-ingestive effects of fescue toxicosis, leading to reduced weight gain and increased core temperatures. Several studies have documented decreased dry matter intake in animals affected by fescue toxicosis [55,56,57], resulting in lower weight gain [57,58].

Prolactin plays a crucial role in the shedding of winter coats, as animals respond to increased day length with elevated prolactin levels [59]. While animals typically shed their winter hair coats during summer, fescue toxicosis reduces serum prolactin concentrations. This reduction occurs due to the ergot alkaloids in WE tall fescue acting as agonists to the D2 dopamine receptors that regulate prolactin [60]. Consequently, this inhibits the shedding of winter hair coats [57,61] and may even increase the growth of summer hair coats [62]. This may have resulted in a tendency towards greater hair coat scores in steers on the LPEG diet, which may have been reduced in steers on the LES diet due to the effect of CTs on ergot alkaloids. Although prolactin levels were not measured in our study, previous research has consistently reported low prolactin levels in cattle exhibiting fescue toxicosis symptoms [63,64].

Steers on the LES diet supplement exhibited lower rectal temperatures compared to steers on LPEG, whereas those on LPEG showed reduced extremity temperatures in comparison to the LES group. A study by Villalba et al. [26] also reported a decrease in rectal temperature in lambs grazing WE tall fescue and consuming sainfoin (2.9% CT), in contrast to lambs consuming cicer milkvetch (*Astragalus cicer*), a non-CT-producing legume. Because the toxic alkaloids in WE tall fescue function as vasoconstrictors, they diminish peripheral blood flow and impair the capacity for heat dissipation [65]. Consequently, animals experience increased internal body temperatures, leading to heat stress. Multiple studies have reported elevated internal core body temperatures due to fescue toxicosis [55,66,67]. The constriction of blood flow to extremities usually results in cooler extremity temperatures in animals affected by fescue toxicosis compared to healthy animals. Numerous studies have observed reduced extremity temperatures in response to toxic ergot alkaloids in tall fescue [43,68,69].

Cortisol, a hormone produced by the adrenal glands, is rapidly released from the adrenal cortex in response to acute stress [70], but circulating cortisol can be elevated in response to chronic stress as well. Fescue toxicosis induces heat stress, and multiple studies have demonstrated elevated cortisol levels in animals experiencing heat stress [44,71,72,73]. Hair cortisol offers insights into long-term chronic stress levels, as it is little influenced by activities such as animal handling [74,75]. Previous research has shown a positive relationship between hair cortisol levels and stress [76,77,78], making hair cortisol a reliable and less invasive indicator of long-term chronic heat stress, particularly in extensive grazing systems [43,44,75,79]. In this study, steers on LPEG exhibited higher hair cortisol levels than those on LES, indicating greater chronic stress in LPEG-fed steers.

The CT-based diet supplement treatment also resulted in a larger luminal area of the caudal artery in the single-season measurements. These results indicate that supplementation with SL pellets for animals grazing toxic fescue can favorably affect their vascular dimensions (although rectal temperatures for both treatments were greater than normal (38.3 °C)). Vasoconstriction is a significant contributor to heat stress and other physiological disruptions associated with fescue toxicosis [80]. The greater caudal artery luminal area found in the LES treatment suggests that supplementing diets with SL pellets helped mitigate vasoconstriction and enhance blood flow. This would help reduce the adverse post-ingestive effects associated with fescue toxicosis by improving thermoregulation, nutrient transport, and overall vascular health. While our study used caudal artery luminal area as a measure of vascular function, the specific pathways through which CTs modulate vascular responses remain to be determined.

## 5. Conclusions

The prevalence of a toxic alkaloid-producing endophytic fungus in tall fescue continues to present challenges to the US beef industry and results in substantial economic losses. The supplementation of feed with CTs presents a potential opportunity to mitigate these detrimental effects. This study indicated that, for steers grazing on WE tall fescue pastures, diet supplementation with CTs led to reduced vasoconstriction, which supported greater extremity temperatures and moderated core body temperatures; in turn, this enhanced weight gain and reduced circulating cortisol. While these results are promising, the mechanisms behind these effects are not clear. Thus, further research is needed to elucidate the mechanisms underlying these effects and establish the long-term efficacy of CT supplementation in animals.

## Figures and Tables

**Table 1:** Average forage biomass (kg ha^−1^) and quality (g kg^−1^ DM) of the experimental pasture across all summers at the Shenandoah Valley Agriculture Research and Extension Center, Raphine, VA, USA.

Year	Forage Measures ^1^				
	Biomass, kg ha^−1^	CP	ADF	NDF	TEA (ppb)
		g kg^−1^ DM			
2021	3910 ± 295.1	103 ± 7.3	376 ± 8.9	690 ± 8.9	1650 ± 333.7
2022	4420 ± 212.4	102 ± 3.8	391 ± 6.4	690 ± 4.7	2330 ± 146.4
2023	4200 ± 327.9	93 ± 10.2	359 ± 7.7	607 ± 9.6	2460 ± 159.7

^1^ CP, crude protein; ADF, acid detergent fiber; NDF, neutral detergent fiber; TEA, total ergot alkaloid.

**Table 2:** Nutritive value (g kg^−2^ DM) of sericea lespedeza pellets, analyzed as single composite samples collected annually from 2021 to 2023.

Year	Nutritive Value, g kg^−1^ DM ^1^		
	CP	ADF	NDF
2021	155	384	466
2022	133	372	453
2023	160	386	416

^1^ CP, crude protein; ADF, acid detergent fiber; NDF, neutral detergent fiber; CT values for the years 2022 and 2023 came from the same pooled source.

**Table 3:** Average daily gain (kg d^−1^) and hair coat score of steers on toxic fescue, supplemented with lespedeza pellets alone (LES) or with LES–polyethylene glycol (LPEG), across three summers.

Measures	Treatments ^1^			
	LES	LPEG	SE	*p*-Value
ADG ^2^, (kg d^−1^)	0.60	0.48	0.032	0.0999
Hair Coat Score	3.85	4.19	0.121	0.0547

^1^ Treatments: LES (sericea lespedeza pellets) and LPEG (sericea lespedeza pellets mixed with polyethylene glycol), supplemented at 0.5% of body weight per day; six fall-born Angus steers assigned to each treatment. ^2^ No significant year × treatment interaction for ADG (*p* = 0.1648) and hair coat score (*p* = 0.0876).

**Table 4:** Extremity and rectal temperatures (°C) of steers on toxic endophyte-infected tall fescue supplemented with either sericea lespedeza pellets (LES) or sericea lespedeza pellets mixed with polyethylene glycol (LPEG) across three summers.

Measures, °C	Treatments ^1^			
	LES	LPEG	SE	*p*-Value
Ear Skin Temperature ^2,3^	28.3	27.9	0.21	0.1826
Hoof Surface Temperature	27.8	27.7	0.25	0.6723
Tail Skin Temperature	27.5	26.6	0.20	0.0053
Rectal Temperature	39.4	39.8	0.04	<0.0001

^1^ Treatments: LES (sericea lespedeza pellets) and LPEG (sericea lespedeza pellets mixed with polyethylene glycol), supplemented at 0.5% of body weight per day; six fall-born Angus steers assigned to each treatment. ^2^ Extremity temperatures determined using a FLIR T630SC thermal camera (Teledyne FLIR LLC, Santa Barbara, CA). ^3^ No significant year × treatment interaction for extremity and rectal temperatures (*p* > 0.1230).

## Data Availability

The data that support the findings of this study are available from the corresponding author on request.

## Supplementary Materials

The following supporting information can be downloaded at https://www.mdpi.com/article/10.3390/ani15030373/s1: Experimental procedures detailing the isolation and purification of the CT reference standard, the composition and structure determination of this CT sample by two-dimensional nuclear magnetic resonance spectroscopy, and the determination of the CT content of the Lespedeza cuneate pellets are provided in the Supplementary Materials accompanying this manuscript.
