# Supplementary material for: Effects of Sericea Lespedeza Supplementation on Steers Grazing Wild-Type Endophyte-Infected Tall Fescue
Source: Animals (Basel). 2025 Jan 28;15(3):373. doi: 10.3390/ani15030373 (PMC11816162; doi:10.3390/ani15030373)
Supplement: Supplementary file 1 [file animals-15-00373-s001.zip › animals-3410437-supplementary.pdf]

Supplemental Materials

Table of Contents

Purification of the *Lespedeza cuneata* Condensed Tannins (CT) Reference Standard..…………………………2

NMR Spectroscopy……………………………………………………………………………………..…………2

Figure S1. ^{1}H-^{13}C HSQC NMR spectrum of the *Lespedeza cuneata* CT
reference standard with signals identified as listed in **Figure S2…**…………………………...………………..3

Figure S2. The structure of a condensed tannin with C-H bonds labeled
corresponding to those listed in **Figure S1…**……………………………………………………...………….3

Composition and structure determination of CT reference standard…………………………………………..4

Purity Assessment of the *Lespedeza cuneata* CT reference standard**…**……………………….…………………4

Figure S3. ^{1}H-^{13}C HSQC NMR spectrum of the *Lespedeza cuneata* CT reference
standard showing the cross-peak signals used to assess the purity of the sample.
The cross-peak signals for the terminal C-4 H/C (Area 1a), the C-4 H/C
A-type interflavan-3-ol linkages (Area 2a), the C-4 H/C B-type interflavan-3-ol
linkages (Area 3a), the C-1 H/C alpha and beta anomeric signals (Area 4a)
and the H/C cross-peaks arising from the carbon-carbon olefin double bonds
in the lipid impurities present (Area 5a) are highlighted……………………………….……………………4

Preparation of HCl-butanol-acetone-iron (HBAI) assay solution……………………………….……………4

Condensed tannin content determinations……………………………………………………………..…………5

Figure S4. The *Lespedeza cuneata* CT reference standard curve…………………………...…………………..5

Deviations from the published procedure…………………………………………………..…………………5

Table S1: Percent CT present in *Lespedeza cuneata* samples 1-4……………………………….…………………6

Acknowledgement…………………………………………………………………………..…………………6

References…………………………………………………………………………………………….…………………6

Purification of the *Lespedeza cuneata* Condensed Tannins (CT) Reference Standard. The *Lespedeza cuneata* CT reference standard was obtained following the procedures described in Brown et al., [1] and Naumann et al. [2]. Ground *Lespedeza cuneata* pellets (25 g) were placed in a 500 mL Erlenmeyer flask equipped with a magnetic stir bar and diluted with acetone/water (7:3, 250 mL). The mixture was rapidly stirred for 30 min and then filtered through a glass-sintered funnel equipped with a filter paper (Reeve Angel, grade 202). The residue was returned to the Erlenmeyer flask and stirred with fresh acetone/water (7:3, 250 mL) and filtered two additional times. The combined three acetone/water extracts were concentrated under reduced pressure (rotary evaporation) at ≤ 40 °C to remove the acetone and the resulting aqueous layer was stirred with ethyl acetate (200 mL) overnight. The aqueous layer was separated using a separatory funnel and stirred a second time with ethyl acetate (200 mL) for about 2 h. The aqueous layer was separated and placed under reduced pressure (rotary evaporation) at ≤ 40 °C to remove any traces of ethyl acetate and then freeze-dried to give 8.5 g of extract. This extract was diluted with methanol/water (1:1, 200 mL) and Sephadex LH-20 (GE Healthcare, Uppsala, Sweden) in small portions with while stirring with a spatula, until the mixture reached the consistency of wet sand (50.0 g of Sephadex LH-20 added). The CT-laced resin was transferred to a 500 mL sintered-glass funnel equipped with a filter paper (Reeve Angel, grade 202). The resin was suspended in methanol/water (1:1, 250 mL), allowed to stand for ~ 5 min and then vacuum filtered. This methanol/water washing of the resin was repeated 14 additional times. The resin was then suspended in acetone/water (7:3, 250 mL), allowed to stand for ~ 5 min and then vacuum filtered. This acetone/water washing of the resin was repeated three additional times. The four acetone/water washings were combined and concentrated under reduced pressure (rotary evaporation) at ≤ 40 °C to remove the acetone and freeze dried to give 720 mg of an off-white solid. The ^{1}H–^{13}C HSQC NMR spectrum of this sample indicated that a purer CT sample was required. Thus, 700 mg of the residue was dissolved in methanol/water (1:1, 30 mL) and Sephadex LH-20 (GE Healthcare, Uppsala, Sweden) in small portions with while stirring with a spatula, until the mixture reached the consistency of wet sand (8.0 g of Sephadex LH-20 added). The CT-laced resin was transferred to a 125 mL sintered-glass funnel equipped with a filter paper (Reeve Angel, grade 202). The resin was suspended in methanol/water (1:1, 80 mL), allowed to stand for ~ 5 min and then vacuum filtered. This methanol/water washing of the resin was repeated 14 additional times. The resin was then suspended in acetone/water (7:3, 90 mL), allowed to stand for ~ 5 min and then vacuum filtered. This acetone/water washing of the resin was repeated four additional times. The four acetone/water washings were combined and concentrated under reduced pressure (rotary evaporation) at ≤ 40 °C to remove the acetone and freeze dried to give 371 mg of an off-white solid, sufficiently pure to serve as the *Lespedeza cuneata* CT reference standard in this study.

NMR Spectroscopy. ^{1}H, ^{13}C, and ^{1}H–^{13}C HSQC NMR spectra for the purified *Lespedeza cuneata* CT were recorded at 27 °C on a BrukerBiospin DMX-500 (^{1}H 500.13 MHz, ^{13}C 125.76 MHz) instrument equipped with TopSpin 3.5 software and a cryogenically cooled 5 mm TXI $^{1}H/^{13}C/^{15}N$ gradient probe in inverse geometry. Spectra were recorded in DMSO-d_6 and were referenced to the residual signals of DMSO-d_6 (2.49 ppm for ^{1}H and 39.5 ppm for ^{13}C spectra). For ^{1}H–^{13}C HSQC experiments, spectra were obtained using the standard Bruker pulse program hsqcegtpsi. Figure S1 shows the ^{1}H-^{13}C HSQC NMR spectrum of the *Lespedeza cuneata* CT reference standard with signals identified as listed in Figure S2.

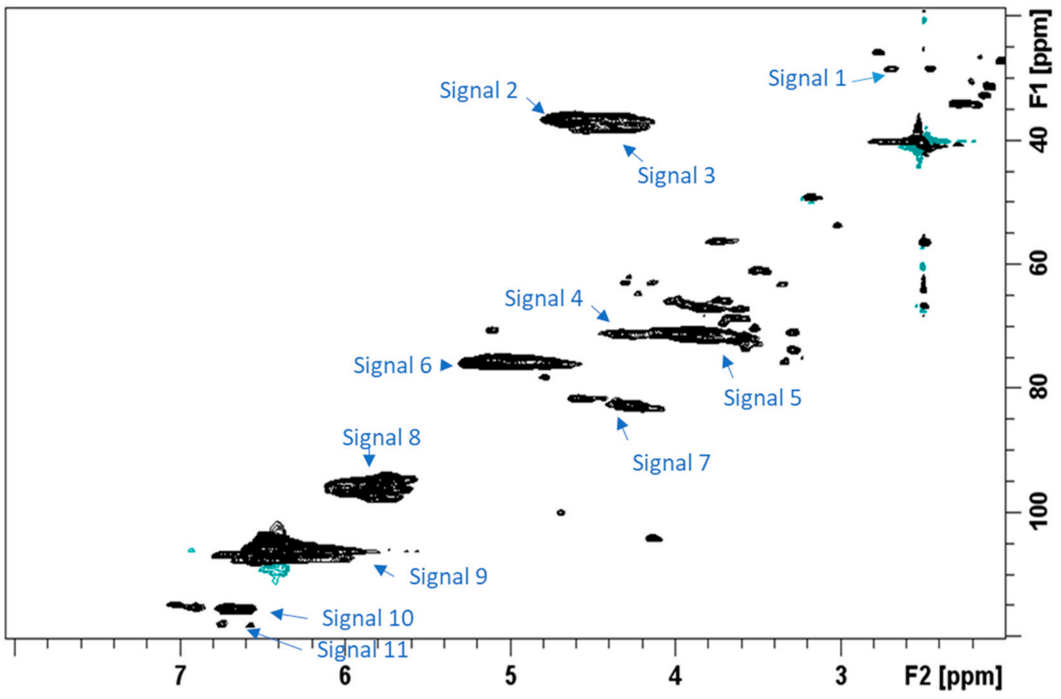

Figure S1. ^{1}H-^{13}C HSQC NMR spectrum of the *Lespedeza cuneata* condensed tannins reference standard with signals identified as listed in **Figure S2**

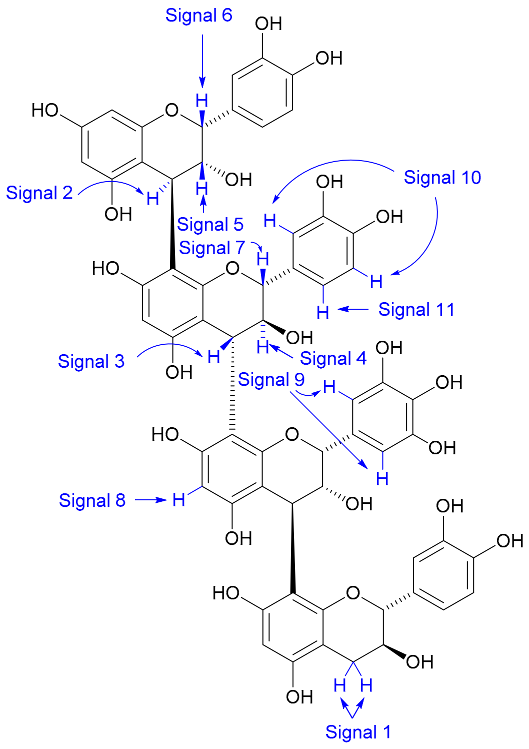

Figure S2. The structure of a condensed tannin with C-H bonds labeled corresponding to those listed in **Figure S1**

Composition and structure determination of CT reference standard. The composition of the CT reference standard was found to have a PC/PD ratio of 2.2/97.8 (± 0.2), a C-2/C-3 *cis/trans* ratio of 78.8/21.2 (± 1.4) [3] and a mean degree of polymerization of 24.2 (± 0.3). In addition to the common B-type linkage present in this reference standard, this sample possessed 3.6 (± 0.1) percent A-type interflavan-3-ol linkages [2].

Purity Assessment of the *Lespedeza cuneata* CT Reference Standard. The purity of the purified *Lespedeza cuneata* CT sample was determined to be approximately 90% based on the relative integration of the impurity NMR cross-peaks arising from carbohydrate anomeric center and olefinic H/C cross-peaks signals versus the C-4 H/C cross peak signals from the CT present (Figure S3).

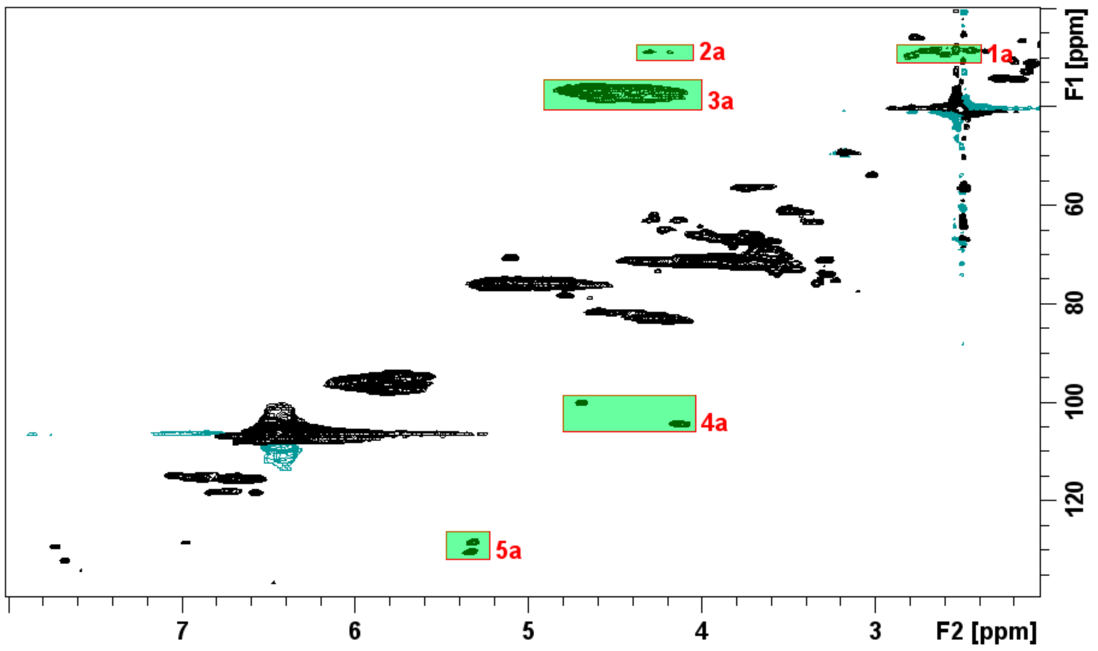

Figure S3. ^{1}H-^{13}C HSQC NMR spectrum of the *Lespedeza cuneata* condensed tannins reference standard showing the cross-peak signals used to assess the purity of the sample. The cross-peak signals for the terminal C-4 H/C (Area 1a), the C-4 H/C A-type interflavan-3-ol linkages (Area 2a), the C-4 H/C B-type interflavan-3-ol linkages (Area 3a), the C-1 H/C alpha and beta anomeric signals (Area 4a) and the H/C cross-peaks arising from the carbon-carbon olefin double bonds in the lipid impurities present (Area 5a) are highlighted

Total condensed tannin content of samples 1-4 were analyzed using the method outlined by Grabber et al. [4].

Preparation of HCl-butanol-acetone-iron (HBAI) assay solution. To a 200 mL volumetric flask equipped with a magnetic stir bar was added ammonium iron (II) sulfate dodecahydrate (300.0 mg, 0.1659 mmol) and water (6.6 mL). Concentrated HCl (10 mL, 12 M) was then added slowly and the mixture was gently stirred for 30 min to ensure dissolution of the iron reagent. The magnetic stir bar was removed from the solution and 1-butanol (60 mL) was added, swirled to mix, followed by acetone (100 mL) and swirled to provide a homogenous solution. The volumetric flask was then diluted to the mark with 1-butanol,

capped and inverted several times to accomplish thorough mixing. The recipe for the assay solution may be scaled up to larger quantities as required.

Condensed tannin content determinations. Condensed tannin content determinations were performed in triplicate. Approximately 10 mg (to the nearest tenth of a mg) of *Lespedeza cuneata* samples were weighed into 25 mL screw cap test tubes. Additional 25 mL screw cap test tubes were charged with CT reference standard solutions (20, 40, 60, 80, 100 and 120 μL) of a 5.00 mg/mL solution in methanol.

The HBAI assay solution (15.0 mL) was added to each test tube using a 15 mL volumetric pipette. The screw caps were added to the test tubes and the tubes were heated in an aluminum block at 70 °C for 3 h. Every 15 min the tubes were removed and briefly vortexed to ensure proper mixing during the reaction.

After cooling to room temperature over about 1 h, 100, 200 and 400 μL aliquots were removed from each of the triplicate runs, placed in 2 mL conical polypropylene-copolymer microcentrifuge tubes, diluted to 2.00 mL through the addition of fresh HBAI assay solution, sealed with screw caps, and centrifuged for 5 min at 10 000 g. After centrifugation, the clarified supernatants were scanned with a Shimadzu UV-2600 spectrophotometer (Shimadzu Scientific Instruments, Columbia, MD) using the Shimadzu UVProbe version 2.43 software package from 400 to 600 nm, and the maximal absorption at λ_{max} of the anthocyanidin peak was recorded. The absorbance data was corrected for the small dilution factor of the reference standards and for the purity of the reference standard. The reference standard curve used for calculating CT content is shown in Figure S4.

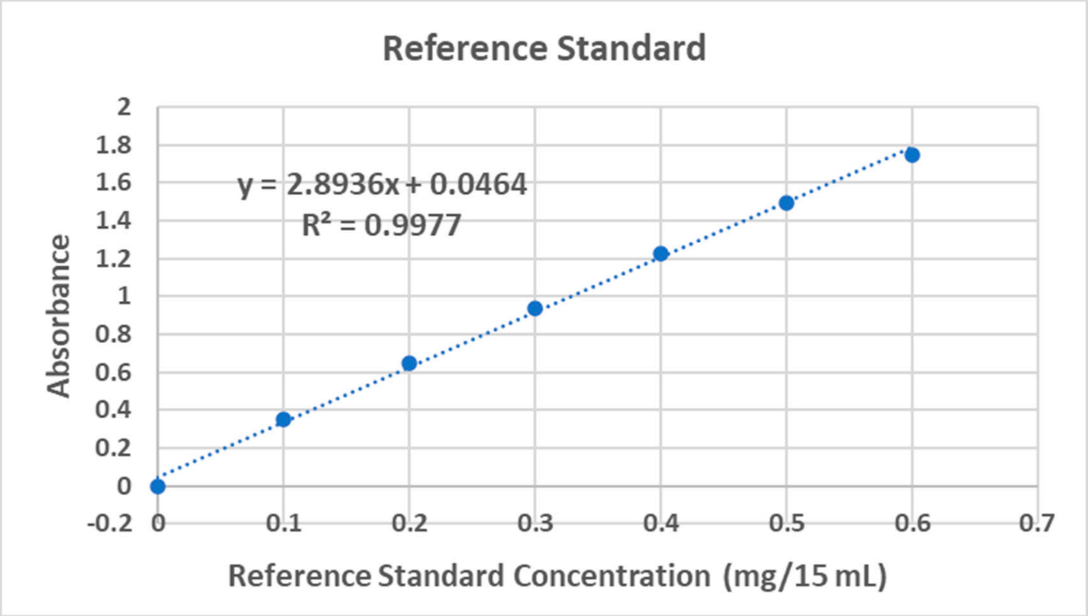

Figure S4. The *Lespedeza cuneata* condensed tannins reference standard curve

Deviations from the published procedure. Due to their high CT content, these *Lespedeza cuneata* samples were handled somewhat differently than previously described [4]. In the direct method for CT content determination, only 10 mg was weighed (to the nearest tenth of a mg) into the 25 mL screw-cap test tubes as opposed the 30 mg for plant materials with lower total CT content as outlined in Grabber et al. [4].

Second, to avoid truncation of the absorbance reading of the *Lespedeza cuneata* samples, the reaction solutions were diluted 5-, 10- and 20-fold before absorbance measurements were taken to ensure absorbance truncation would be circumvented. CT content from samples 1-4 are given in Table 1.

Table S1: Percent CT present in *Lespedeza cuneata* samples 1-4.

Dilution	Sample 1 (2021)	Sample 2 (2022-1)	Sample 3 (2022-2)	Sample 4 (2022-3)
1/5 dilution	19.0 (2.5)	20.7 (1.9)	20.7 (0.2)	21.5 (0.7)
1/10 dilution	19.3 (1.3)	20.5 (1.1)	20.2 (0.4)	21.4 (1.1)
1/20 dilution	18.3 (0.6)	19.9 (0.4)	18.4 (1.1)	20.7 (1.7)
%wt CT (STDEV)<br>Values are averages of triplicate runs.				

Acknowledgement: The mention of trade names or commercial products in this article is solely for the purpose of providing specific information and does not imply recommendations or endorsements by the U.S. Department of Agriculture. The USDA is an equal opportunity provider and employer.

References

1. Brown, R.H.; Mueller-Harvey, I.; Zeller, W.E.; Reinhardt, L.; Stringano, E.; Gea, A.; Drake, C.; Ropiak, H.M.; Fryganas, C.; Ramsay, A. Facile purification of milligram to gram quantities of condensed tannins according to mean degree of polymerization and flavan-3-ol subunit composition. *J. Agric. Food Chem* **2017**, *65*, 8072-8082.
2. Naumann, H.; Sepela, R.; Rezaire, A.; Masih, S.E.; Zeller, W.E.; Reinhardt, L.A.; Robe, J.T.; Sullivan, M.L.; Hagerman, A.E. Relationships between structures of condensed tannins from Texas legumes and methane production during in vitro rumen digestion. *Molecules* **2018**, *23*, 2123.
3. Zeller, W.E.; Ramsay, A.; Ropiak, H.M.; Fryganas, C.; Mueller-Harvey, I.; Brown, R.H.; Drake, C.; Grabber, J.H. 1H–13C HSQC NMR spectroscopy for estimating procyanidin/prodelphinidin and cis/trans-flavan-3-ol ratios of condensed tannin samples: Correlation with thiolysis. *J. Agric. Food Chem* **2015**, *63*, 1967-1973.
4. Grabber, J.H.; Zeller, W.E.; Mueller-Harvey, I. Acetone enhances the direct analysis of procyanidin-and prodelphinidin-based condensed tannins in Lotus species by the butanol–HCl–iron assay. *J. Agric. Food Chem* **2013**, *61*, 2669-2678.
